# DUF916 and DUF3324 in the WxL protein cluster bind to WxL and link bacterial and host surfaces

**DOI:** 10.1002/pro.4806

**Published:** 2023-10-25

**Authors:** Mahreen U. Hassan, Roy R. Chaudhuri, Mike P. Williamson

**Affiliations:** ^1^ School of Biosciences University of Sheffield Sheffield UK; ^2^ Present address: Department of Microbiology Shaheed Benazir Bhutto Women University Peshawar Pakistan

**Keywords:** AlphaFold2, peptidoglycan, structure prediction, virulence, WxL

## Abstract

Bacterial WxL proteins contain peptidoglycan‐binding WxL domains, which have a dual Trp‐x‐Leu motif and are involved in virulence. It was recently shown that WxL proteins occur in gene clusters, containing typically a small WxL protein (which in the mature protein consists only of a WxL domain), a large WxL protein (which contains a C‐terminal WxL domain with N‐terminal host‐binding domains), and a conserved protein annotated as a Domain of Unknown Function (DUF). Here we analyze this DUF and show that it contains two tandem domains—DUF916 and DUF3324—which both have an IgG‐like fold and together form a single functional unit, connected to a C‐terminal transmembrane helix. DUF3324 is a stable domain, while DUF916 is less stable and is likely to require a stabilizing interaction with WxL. The protein is suggested to have an important role to bind and stabilize WxL on the peptidoglycan surface, via the DUF916 domain, and to bind to host cells via the DUF3324 domain. AlphaFold2 predicts that a β‐hairpin strand from DUF916 inserts into WxL adjacent to its N‐terminus. We therefore propose to rename the DUF916‐DUF3324 pair as WxL Interacting Protein (WxLIP), with DUF916, DUF3324 and the transmembrane helix forming the first, second and third domains of WxLIP, which we characterize as peptidoglycan binding domain (PGBD), host binding domain (HBD), and transmembrane helix (TMH) respectively.

## 1 INTRODUCTION

WxL proteins have been identified as cell surface proteins in a wide distribution of firmicutes, almost entirely in the orders Lactobacillales and Bacillales, which contain gut commensal organisms such as *Enterococcus faecalis*, *Enterococcus faecium*, *Listeria monocytogenes*, and *Lactiplantibacillus* (formerly *Lactobacillus*) *plantarum*. It is a domain of 160–190 amino acids containing two conserved motifs with the sequence Trp‐x‐Leu (Galloway‐Peña et al., 2015). We recently carried out a bioinformatics study of WxL proteins (Hassan & Williamson, 2023), and showed that they occur within a gene cluster that typically contains a small WxL protein, a large WxL protein, and a Domain of Unknown Function (DUF). Small WxL proteins contain only the WxL domain, plus a signal peptide, while large WxL proteins contain a WxL at the C‐terminus, preceded by between one and four other domains, many of which have functions in binding to host epithelial cells or evading immune detection (Brinster et al., 2007; Cortes‐Perez et al., 2015; Galloway‐Peña et al., 2015; Nunez et al., 2018; Hassan & Williamson, 2023), which fits the profile of WxL proteins as having a role in virulence (Bourgogne et al., 2008; Solheim et al., 2011; Castro et al., 2017; Jamet et al., 2017). We showed that the WxL domain binds to peptidoglycan, and suggested that the WxL cluster consists of a platform of two WxL domains, which bind to peptidoglycan and anchor the protein cluster onto the bacterial surface, from which protrudes a number of other domains that bind to eukaryotic hosts or aid in immune escape.

Our bioinformatic analysis (Hassan & Williamson, 2023) suggested that the DUF protein that is an essential component of the gene cluster must also be involved. This work therefore presents an analysis of the structure and function of the DUF protein, using a combination of bioinformatics and experimental work. The Pfam database contains 4000 DUF families, of which only 22% have a suggested function (El‐Gebali et al., 2019). A study reported that 20% of proteins are annotated as DUF proteins (Bateman et al., 2010). There is thus a need to define the core functions of DUF domains. This work goes a small way towards that aim, by defining the function of the DUF916/DUF3324 pair as being a single unit that binds peptidoglycan (via DUF916), interacts with the eukaryotic host (via DUF3324 and the transmembrane helix that follows it) and stabilizes the WxL platform, by interacting with a WxL domain. We therefore propose that the DUF916‐DUF3324‐TM protein be renamed as WxL Interacting Protein (WxLIP), with the constituent DUF916, DUF3324 and TM domains being named peptidoglycan binding domain (PGBD), host binding domain (HBD) and transmembrane helix (TMH) respectively. Earlier literature has used confusing and sometimes contradictory names: in this paper we are consistent in using the new names.

## 2 RESULTS

### 2.1 Domain analysis

We start by analyzing the DUF proteins from the well characterized WxL clusters from the human symbionts *L. plantarum WCFS1* (*Lp*WxLIP1–9), *L. monocytogenes* (*Lm*WxLIP1 and *Lm*WxLIP2), *E. faecium DO* (*Efm*WxLIP1, 2, and 3), and *E. faecalis* V583 (*Efs*WxLIP) (Table S1), and from this group extend to cover a wide sample of bacterial DUF916 and DUF3324 domains.

**FIGURE 1:**
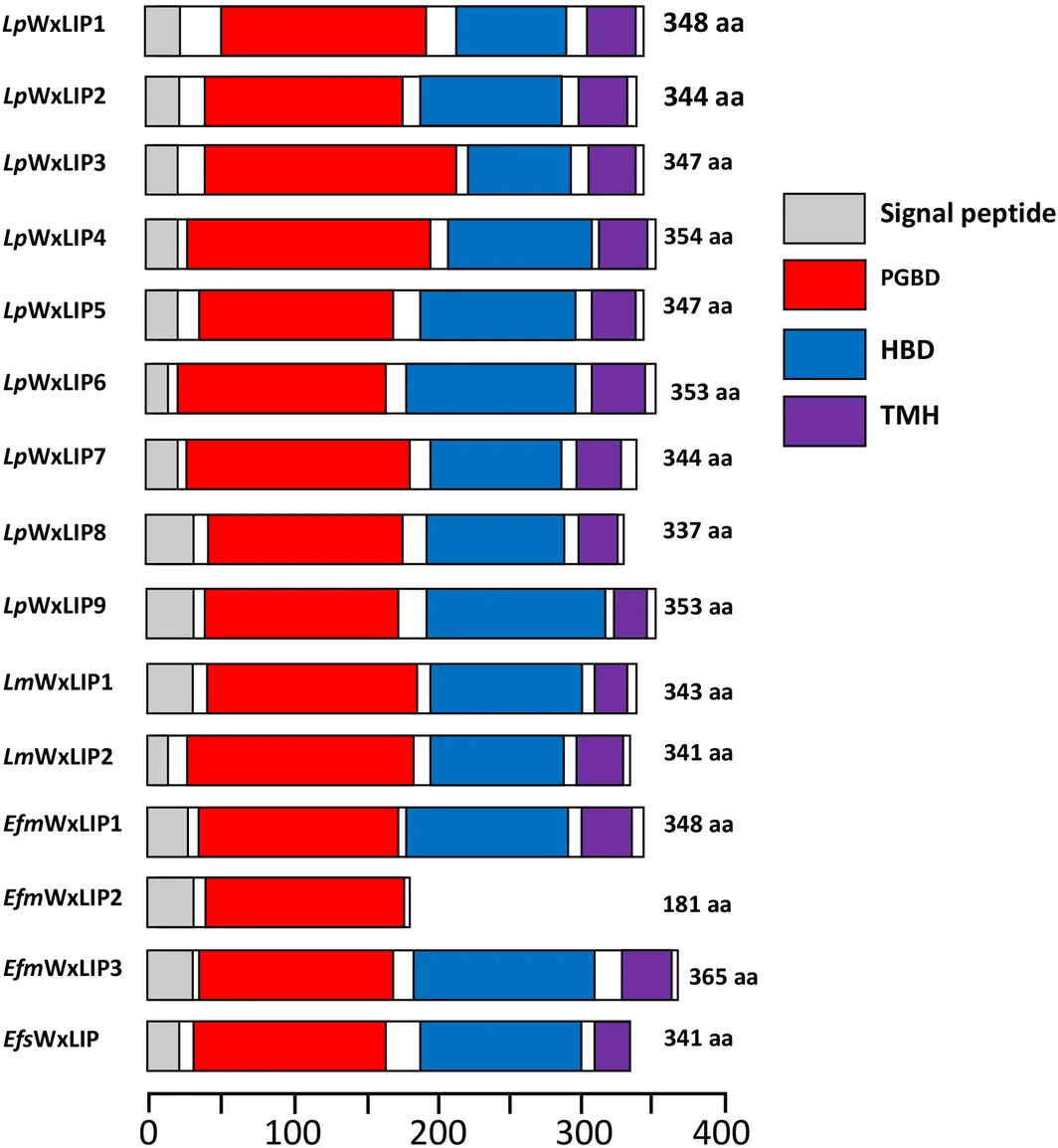
Schematic representation of WxLIP proteins. *Lp*WxLIP1–*Lp*WxLIP9 are from *L. plantarum* WCFS1 (Cluster 1–9); *Lm*WxLIP1 and *Lm*WxLIP2 are from *L. monocytogenes* cluster 1 and 2, respectively; *Efm*WxLIP1–3 are from *E. faecium* DO Locus A to C; and *Efs*WxLIP is from *E. faecalis* V583.

Bioinformatic tools were used to identify domains, which were then further checked using AlphaFold2. The analyses consistently showed WxLIP proteins to consist of three domains: PGBD, HBD and TMH as shown in Figure 1 and Tables S2–S4, where PGBD corresponds to DUF916, HBD to DUF3324, and TMH to a transmembrane helix. PGBD is approximately 148 amino acids long and HBD is 112 amino acids long according to AlphaFold2. TMH is 22 residues long, consistent with its identification as a transmembrane helix.

We note that the current version of Pfam, which was derived using sequence analysis rather than structural predictions, identifies the DUF916 domain as being approximately 125 residues long. DUF916 only rarely occurs as an isolated domain (not part of the full WxLIP); however, when it does occur alone it is generally of roughly this length. In the context of WxLIP, the PGBD domain contains a 20‐residue insert, which we describe below as a buttressing loop, which functions to restrict the relative orientations of the PGBD and HBD domains within the full‐length WxLIP protein.

The domain structures of the proteins from the WxL clusters are indicated in Figure 1. In this group, the size of the WxLIP proteins ranges from 337 to 365 residues in length with the exception of *Efm*WxLIP2, which was anomalously small at 181 residues in length, and may represent a partial loss of function arising from loss of the HBD and TM domains. Siezen et al. (2006) suggested a slightly larger range of 320–380 residues long. Their molecular weights are between 37 and 42 kDa with the exception of *Efm*WxLIP2, which has a molecular weight of 20.2 kDa. The predicted pI of WxLIP proteins from *L. plantarum WCFS1* and *L. monocytogenes* is highly basic from around 8 to 11 while the proteins from *Enterococcus* species have acidic pIs of 5.1–6.8, as shown in Table S1, in agreement with Siezen et al., who reported that the proteins from *L. plantarum WCFS1* species are predicted to be highly basic proteins (Siezen et al., 2006).

Figure 1 shows that all proteins contain a signal peptide, implying that they are secreted proteins. In agreement with this conclusion, we note that WxL proteins are also secreted, and we previously concluded that all proteins within the WxL gene cluster are bacterial surface proteins (Hassan & Williamson, 2023).

**FIGURE 2:**
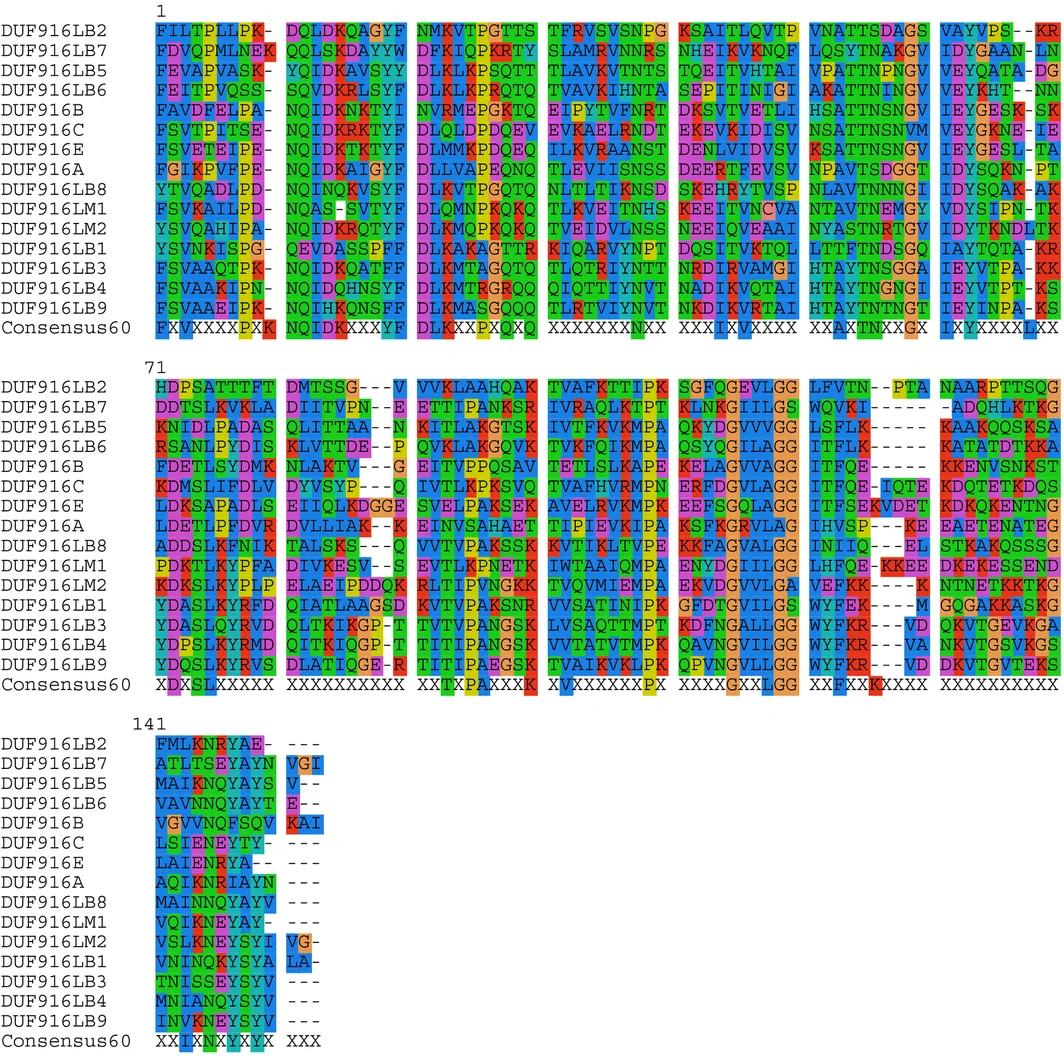
Sequence alignment of PGBD (DUF916) domains from WxL clusters. The figure was produced using MUSCLE (Edgar, 2004), and the visualization used SEAVIEW and PHYLO_WIN.

**FIGURE 3:**
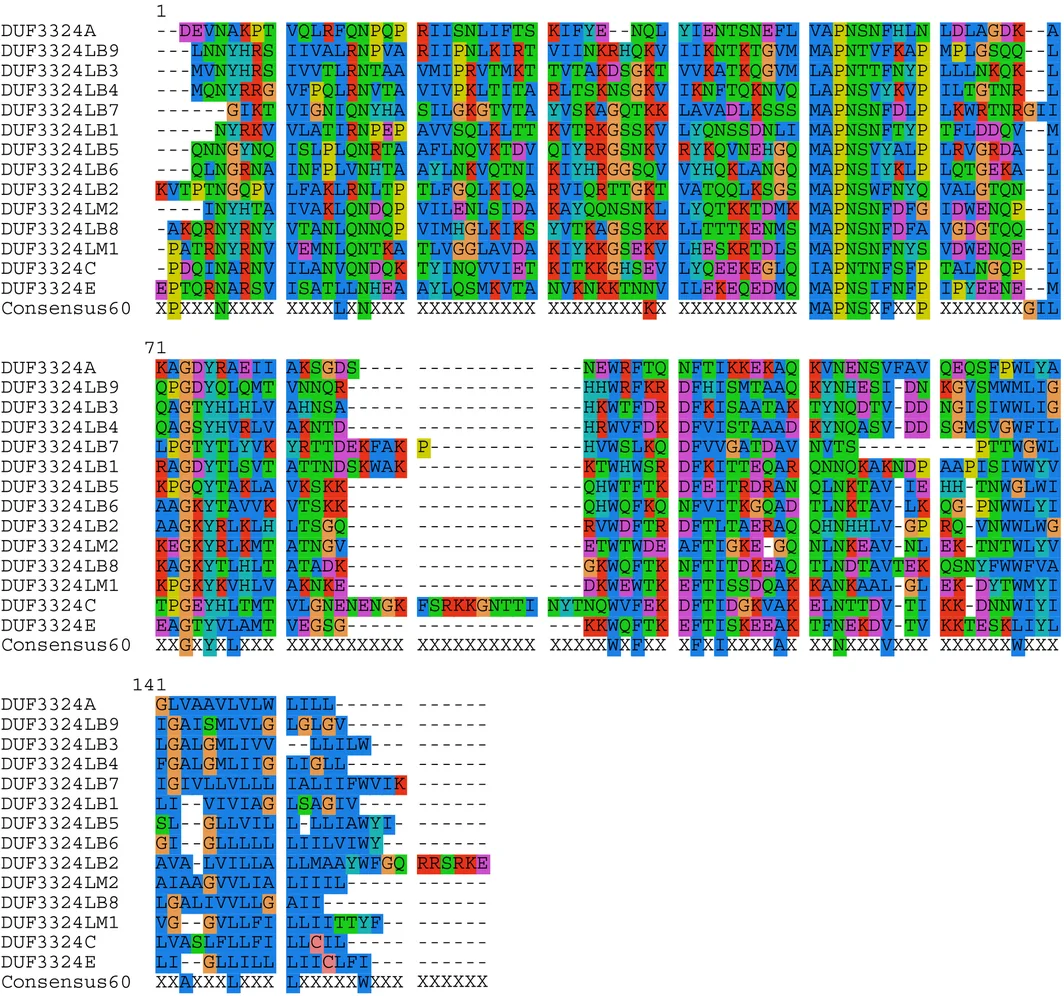
Sequence alignment of the HBD (DUF3324) domains from WxL clusters. The figure was produced using MUSCLE (Edgar, 2004).

Sequence analysis of the PGBD and HBD domains shows the presence of some highly conserved residues (Figures 2 and 3). In particular, PGBD contains a highly conserved sequence NQIDKxxxYFDLK close to the N‐terminus.

### 2.2 Distribution of DUF916 and DUF3324 domains

We then carried out a more comprehensive analysis of the occurrence of WxL and WxLIP proteins in bacterial genomes (Table S5) and of their sequences (Figures S1 and S2). This analysis found 1308 proteins in total, comprising 47 PGBD alone, 25 HBD alone, 512 full‐length WxLIP, and 724 WxL. These domains have a strong tendency to occur together in clusters within the genome: there are only 59 occurrences of a genome that contains only one or two WxL and/or WxLIP proteins (colored blue in Table S5). We defined a WxL cluster as a group of adjacent genes (separated by no more than four annotated genes) that must contain at least one WxLIP and two WxL proteins. On this definition, there are 179 clusters. Of these, 98 contain exactly one WxLIP and two WxL, making this simple arrangement (identified from the clusters shown in Figure 1) the most common type of WxL cluster. In all cases, the WxLIP protein contains both the PGBD and HBD domains. There are only two instances of clusters that contain isolated PGBD or HBD proteins, and both of these clusters also contain a full‐length WxLIP. It is therefore clear that that the association of one WxLIP protein and two WxL proteins into a cluster provides a functional benefit to the cell, presumably arising from a physical interaction between these proteins.

The pairing of the PGBD and HBD domains is highly conserved (Table S5), and the two domains always occur in this order. PGBD domains all contain a 20‐residue insert which is not present in the DUF916 domain definition in Pfam. We therefore conclude that, although single DUF916 or DUF3324 domains may have independent functions, the specific pairing of the two domains seems to be tightly associated with WxL domains: our analysis suggests that the full‐length WxLIP has a specific function that involves association with a WxL domain. Furthermore, we show below that WxLIP interacts in a specific manner with WxL. We therefore propose that the designations DUF916 and DUF3324 are unnecessary and should be replaced by WxLIP (domains PGBD and HBD respectively).

### 2.3 Structures of WxLIP proteins

**FIGURE 4:**
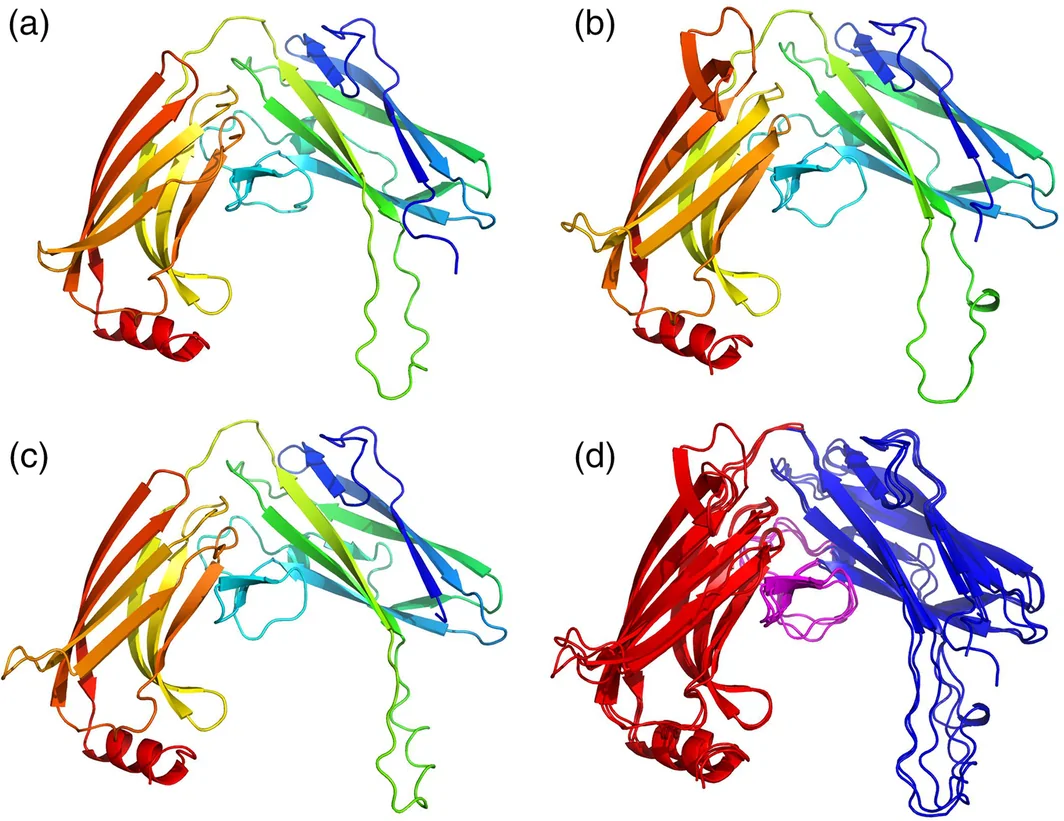
AlphaFold2 structures of WxLIP. (a), (b), and (c) show the structures of the proteins from *E. faecium* cluster A, *E. faecium* cluster C, and *E. faecalis* respectively, using rainbow colors going from blue at the N‐terminus to red at the C‐terminus. (d) is a superposition of the three, with the PGBD domain in blue, the HBD domain in red, and the buttressing loop in maroon, which forms an insert into the PGBD domain. In all cases the TMH domain is predicted to be a long helix projecting out from the C‐terminus and is omitted for clarity.

The structures of WxLIP proteins were predicted using Phyre2, Robetta, and AlphaFold2. All the predictions gave similar results, giving us a high degree of confidence in the predictions. The results for Phyre2 and Robetta are shown in Figures S3 and S4 and Tables S6 and S7. The results for AlphaFold2 are shown in Figure 4. The predicted structures have good geometrical quality (Tables S8–S11).

Both PGBD and HBD are predicted to be β‐sandwich folds, with structural similarity to the Ig fold. The two domains are predicted to be in close contact; interestingly, almost all the predictions place the two domains perpendicular to each other, as shown in Figure 4. The angle between the two domains is maintained by a conserved loop protruding from the PGBD domain, shown in maroon in Figure 4d. This loop constitutes the additional residues within the PGBD domains in the clusters.

**FIGURE 5:**
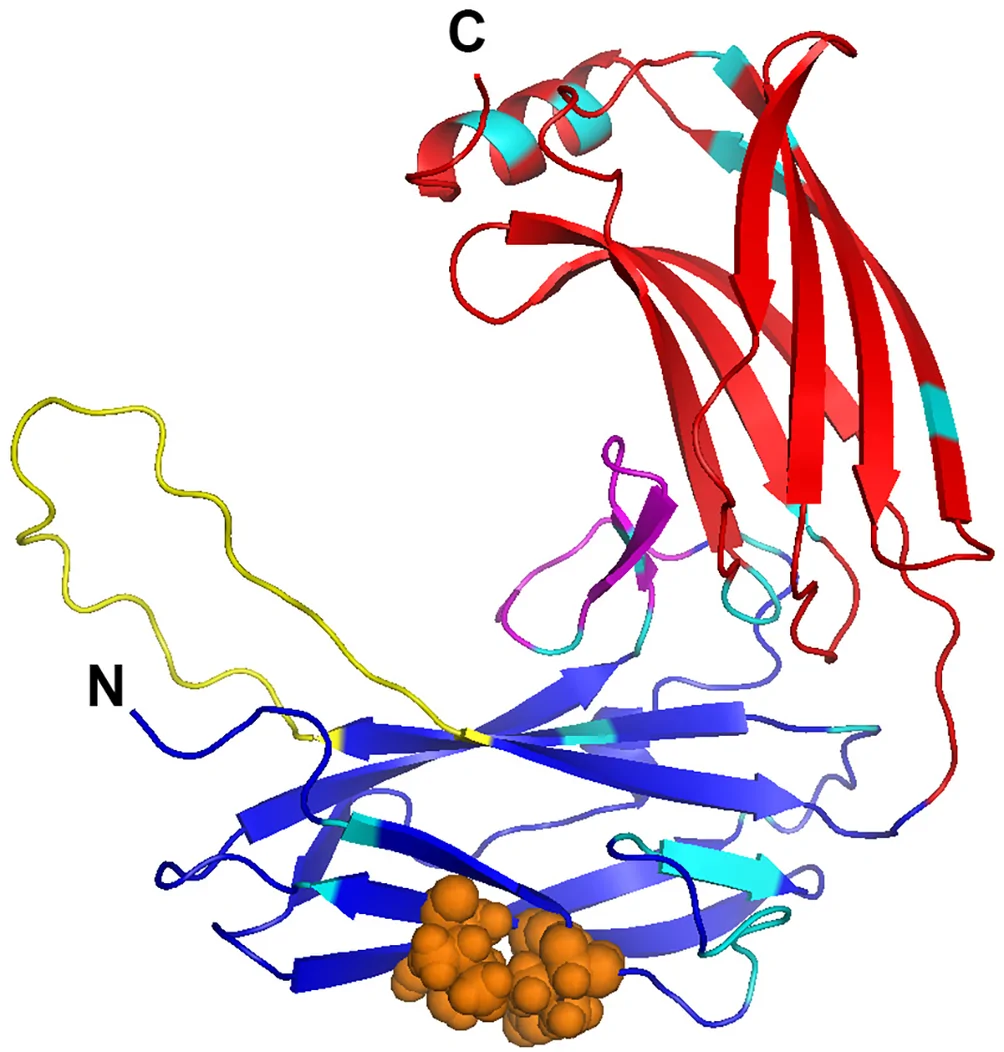
Features of the WxLIP structure, shown using the *Efm*WxLIP1 model from AlphaFold2. The coloring of the backbone is as in Figure 4d: PGBD is blue, the buttressing loop in maroon, and HBD in red. The predicted N‐acetyl glucosamine binding site is shown in orange spheres, and the protein is oriented to put this binding site (and thus by implication the bacterial cell wall) at the bottom of the figure. Conserved residues (red and cyan in Figures 2 and 3) are in cyan. The C‐terminal TMH is not shown. The strand suggested by AlphaFold2 to insert into WxL is shown in yellow.

Conserved residues within WxLIP were identified in Figures 2 and 3 and are indicated in Figure 5. The conserved residues are concentrated in the interface between the two domains; at the top of HBD, which is expected to be the site of interaction with host proteins on the basis of sequence homology to bacterial pilin proteins; at the bottom of PGBD; and in the conserved N‐terminal sequence NQIDKxxxYFDLK. Further evidence that residues at the top of HBD are involved in interactions with host is provided by a comparison of the residues conserved within the human symbiont clusters, as listed in Figures 2 and 3, and the residues conserved among all bacterial genomes (Figures S1 and S2), shown in Figure S5. The two sets of conserved residues are similar, except for the absence of the set of conserved residues at the “top” of HBD, because of the much wider range of hosts in these proteins.

The structure of PGBD (blue in Figure 5) is a classic β sandwich, except that the N‐terminal strand is split into two smaller strands, which occupy a distorted position such that the sandwich is not completed in an optimal manner where it bends over to form the sandwich. The strongly conserved NQIDKxxxYFDLK sequence (in cyan at the bottom right of Figure 5) forms the second smaller strand, suggesting a functional role for this sequence.

### 2.4 Docking predictions

Docking predictions made with 3D‐ligand are shown in Table S12, and indicate that almost all WxLIP proteins are predicted to interact with N‐acetyl glucosamine, which is the main saccharide building block of peptidoglycan. The interaction is mainly but not exclusively predicted to be via the PGBD domain. Given that the WxL cluster is located on the bacterial surface, and WxL domains are expected to bind peptidoglycan, this is not an unexpected result.

Protein partners of WxLIP were predicted using the STRING webserver. The analysis was reported in part in our previous publication (Hassan & Williamson, 2023) and indicates that WxLIP interacts with WxL domains. This is again not a surprise, given that the WxL cluster is defined as being composed of WxL and WxLIP proteins. The binding site for peptidoglycan is illustrated by orange spheres in Figure 5.

**FIGURE 6:**
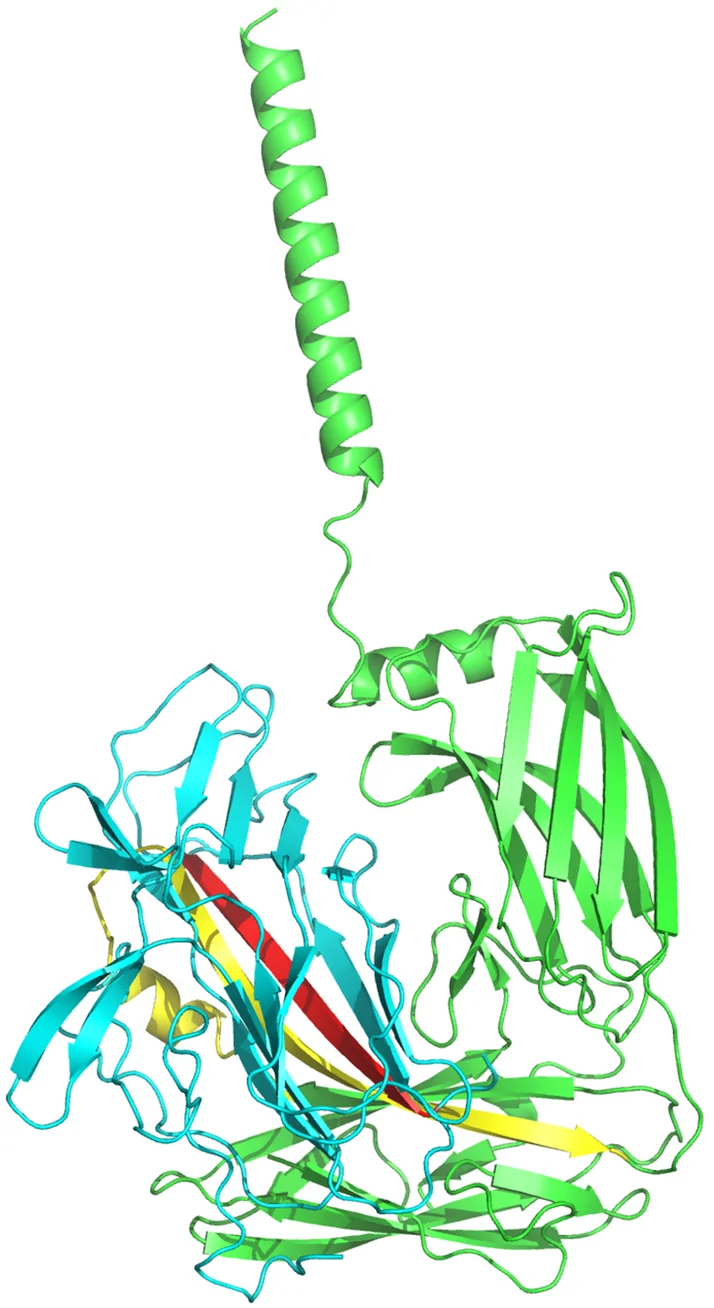
AlphaFold2 prediction for the complex between *Efm*WxLIP1 and WxL from the same gene locus. WxLIP is in the same orientation as in Figure 5, though now showing the C‐terminal TMH at the top of the Figure. WxL is in cyan. The WxLIP hairpin loop (yellow) has become more ordered, forming a long strand parallel to a WxL strand (red).

AlphaFold2 was used to predict structures of complexes between WxLIP and WxL, using the ColabFold server (Mirdita et al., 2022). The predictions were confident and consistent. In all cases, the long hairpin loop in WxLIP (yellow in Figure 5), which is lacking in secondary structure in the AlphaFold prediction of WxLIP, becomes ordered and makes a parallel β‐sheet interaction with a long strand in WxL. The newly structured WxLIP β‐strand fits into a gap in WxL (Figure 6) to complete the WxL β‐sandwich.

### 2.5 Expression and stability of *E. faecium* *Efm*WxLIP3

We set out to test some of these predictions experimentally, in particular the suggestion that PGBD requires additional stabilization by interaction, while HBD is independently stable. All the WxLIP proteins discussed here have similar sequence and domain structure. We therefore focused on one typical example, namely *Efm*WxLIP3 from *E. faecium* locus C. This is a simple locus, predicted to contain only a short WxL, a long WxL and a WxLIP protein (Hassan & Williamson, 2023). No experimental work has previously been done on *Efm*WxLIP3.

A multiple sequence alignment was made of the WxLIP proteins from locus A, B, C of *E. faecium* DO and *E. faecalis* V583 species, shown in Figure S6. Based on the alignment, three constructs were designed. They were made by Gibson assembly (Gibson et al., 2009), and consisted of three synthetic G‐block sequences coding for PGBD, PGBD and HBD together, and HBD, which were inserted into a linear pOPINF plasmid and designated pDUF916_1, 2, and 3 respectively (Figure S7). The coding sequence ended near the start of the transmembrane domain.

The three different constructs were each expressed in different *E. coli* cells: BL21(DE3), BL21 gold(DE3), and BL21(DE3) lemo. Each transformation was grown at three different temperatures, for three different times with three different concentrations of IPTG for induction. The BL21(DE3) lemo cells showed best expression. pDUF916_3 (the construct encoding HBD) showed good expression at 37°C using 1 mM IPTG for 4 h, and was found in the supernatant, while the other two constructs showed optimal expression at 25°C with 0.5 mM IPTG overnight, although at much lower levels (Figure S8), with the majority of the protein occurring in the pellet. The presence of each protein was confirmed by mass spectrometry after tryptic digest (Table S13). The amount of HBD (pDUF916_3) was considerably more than either of the constructs containing PGBD (pDUF916_1 and pDUF916_2). It is concluded that the HBD domain is independently stable and soluble, but PGBD has much lower solubility, either alone or in combination with HBD.

## 3 DISCUSSION

The results of the analysis presented here are consistent, and strongly imply that WxLIP is a single functional unit, in which the PGBD domain binds to peptidoglycan and to WxL, HBD points out from the surface and is likely to interact with proteins on the surface of host endothelial cells, and TMH is a transmembrane helix.

The structures shown in Figures 4, 5, 6 suggest that PGBD becomes more ordered when it binds to WxL. Our experimental work confirms this, in that it shows that the *Efm*WxLIP3 HBD domain is stable, whereas *Efm*WxLIP3 PGBD has low stability and solubility and is poorly expressed. We suggest that the interaction between PGBD and WxL (Figure 6) stabilizes both partners. Other authors have also suggested that the interaction between WxL and WxLIP stabilizes WxL and supports its function in virulence (Cortes‐Perez et al., 2015; Galloway‐Peña et al., 2015).

The C‐terminal TMH domain is present in virtually all WxLIP proteins (Figure S2), although there is no clear sequence homology and our analyses suggest that the gene sequences may have been acquired from a wide range of sources. It is therefore likely to have a functional role in inserting into a membrane. We feel that this is very unlikely to be the bacterial cell membrane, because this is inaccessible from the outside of the peptidoglycan layer. HBD interacts with host cell surfaces in the intestinal lining, and we therefore propose that TMH inserts into the host cell membrane. Such an insertion would have a clear role in strengthening the attachment of the bacterial cell, and facilitating virulence, a function linked to the WxL locus.

These proposals are based largely on an in silico analysis and should be confirmed experimentally. To do so requires soluble and stable protein, which could possibly be prepared using a WxL/WxLIP complex. Our experimental efforts are aimed at this goal.

In summary, the separate DUF916 and DUF3342 annotations should be replaced by a single WxL Interacting Protein (WxLIP) annotation, with most WxLIP consisting of three domains: PGBD, HBD and TMH. We suggest that it has a role in anchoring WxL onto the peptidoglycan layer and in linking the bacterial cell surface to its host.

## 4 MATERIALS AND METHODS

### 4.1 Protein selections and sequence retrieval

The sequence retrieval was done with the help of Uniprot. The accession numbers of the proteins used in this study are detailed in Table S1.

### 4.2 Protein characteristics

Physicochemical properties were determined with the ProtParam tool available from ExPASy (https://web.expasy.org/protparam) and are listed in Table S1. Protein location, signal peptides, and transmembrane helices were analyzed using CELLO (http://cello.life.nctu.edu.tw/), TargetP 2.0 (http://www.cbs.dtu.dk/services/TargetP/), cNLS Mapper (http://nls‐mapper.iab.keio.ac.jp/cgi‐bin/NLS_Mapper_form.cgi), TMHMM server v. 2.0 (https://services.healthtech.dtu.dk/service.php?TMHMM‐2.0), HMMTOP (http://www.enzim.hu/hmmtop/html/submit.html), and Protter (https://wlab.ethz.ch/protter/start/). Interaction networks were identified using the STRING database (https://string-db.org) with a score cut‐off value of 0.40.

### 4.3 Domain and fold analysis

Domains, motifs and families were identified using InterProScan (https://www.ebi.ac.uk/interpro/result/InterProScan/), Conserved Domain Architecture Retrieval Tool (CDART) (https://www.ncbi.nlm.nih.gov/Structure/lexington/lexington.cgi), Simple Modular Architecture Research Tool (SMART) (http://smart.embl-heidelberg.de/smart/show_motifs.pl), BlastP (https://blast.ncbi.nlm.nih.gov/Blast.cg), Motif finder (https://www.genome.jp/tools/motif/), and the PFP‐FunDSeqE predictor web server. InterProScan allows the scanning of sequences against the InterPro signatures collected from different databases (Jones et al., 2014). CDART searches protein similarities against the NCBI Entrez Protein Database using profiles of protein domains and scores them based on the domain architecture (Geer et al., 2002). SMART is a resource of manually curated protein domain models, which identifies, annotates and explores the architecture of protein domains (Letunic et al., 2015). Sequence alignments were done using MUSCLE (Multiple Sequence Comparison by log Expectation) (https://www.ebi.ac.uk/Tools/msa/muscle/) (Edgar, 2004) and ClustalW (Madeira et al., 2022) and visualized using SeaView (https://doua.prabi.fr/software/seaview) (Gouy et al., 2010).

A set of 5482 bacterial genomes, each representing a different species, were obtained from GenBank. These were investigated for the presence of the WxL, DUF916, and DUF3324 domains (Pfam accession numbers PF13731.8, PF06040.14, and PF11797.10, respectively) using the hmmsearch tool from HMMER version 3.3.2 (Eddy, 2011). hmmsearch hits with an *E*‐value below 1 × 10^−10^ are included in Table S5, together with information on the gene encoding each protein, obtained from the GenBank record.

### 4.4 Structure prediction

Secondary structure was predicted using PSIPRED (McGuffin et al., 2000). Tertiary structure was predicted using AlphaFold2 (https://alphafold.ebi.ac.uk) (Jumper et al., 2021), Robetta (https://robetta.bakerlab.org/) (Kim et al., 2004), and Phyre2 (http://www.sbg.bio.ic.ac.uk/phyre2/) (Kelley et al., 2015). Protein topology was determined using PDBsum (http://www.ebi.ac.uk/thornton-srv/databases/pdbsum) (Laskowski et al., 2018). The structures produced were analyzed in DALI (Holm, 2020) to identify related structures. Predictions were analyzed using ProCheck, ERRAT, ProSA (Protein Structure Analysis), and Verify3D. The binding pockets were explored by 3D‐Ligand and CastP. Protein interactions were measured by STRING analysis.

### 4.5 Protein expression and purification

Three *Efm*WxLIP3 constructs (Figure S7) were transformed into BL21 lemo(DE3), BL21 gold(DE3) and BL21(DE3) cells for expression of the protein, which were spread on LB agar plates with 100 μg/mL ampicillin. A single transformed colony was selected from the transformed cell plate and was inoculated aseptically into 5 mL LB broth + ampicillin as a starter culture; the flask was then incubated at 37°C overnight. Five hundred microliters of culture was taken from the flask and was added into 50 mL LB broth containing ampicillin and the flask was incubated at 37°C. Every 2 h, the optical density (OD) was checked using a nanodrop (Thermo Scientific Nanodrop). When the OD reached 0.7, IPTG was added to the flask from a 1 M stock to final concentrations of 0.1, 0.5, and 1 mM, and flasks were incubated at three different temperatures (25, 30, and 37°C), and for three different times (3, 4 and 16 h). The cell suspension from each flask was transferred into a Falcon centrifuge tube and spun for 12 min at 8000 rpm at 4°C in a Beckman Avanti J‐251 centrifuge. After the centrifugation, the supernatant was discarded, and the pellet was re‐suspended in 1 mL lysis buffer (50 mM Tris HCl pH 7.0, 250 mM NaCl, 15% Roche complete EDTA free protease inhibitor). The mixture was sonicated and centrifuged at 18,000 rpm for 20 min at 4°C in a Beckman Avanti J‐251 centrifuge. The supernatants were filtered through a 0.45 μm Minisart filter. The supernatant and pellet were then run on a 16% SDS gel. The remaining samples were stored at −20°C.

## AUTHOR CONTRIBUTIONS

**Mahreen U. Hassan**: Investigation; writing‐original draft; writing‐review and editing. **Roy R. Chaudhuri**: Investigation. **Mike P. Williamson**: Funding acquisition; investigation; supervision; writing‐review and editing.

## CONFLICT OF INTEREST STATEMENT

The authors declare that they have no known competing financial interests or personal relationships that could have appeared to influence the work reported in this paper.

## Supporting information


**TABLE S1:** Characteristics of WxLIP proteins from WxL clusters.
**TABLE S2.** Position of different domains within different WxLIP proteins and detected by BlastP analysis and Motif Finder.
**TABLE S3.** Position of different domains within different WxLIP proteins using InterPro, CDART, and SMART analysis.
**TABLE S4.** Position of different domains within different WxLIP proteins by AlphaFold2 analysis.
**TABLE S6.** Phyre2 analysis.
**TABLE S7.** Robetta analysis of *Enterococcus* proteins having WxLIP proteins.
**TABLE S8.** Evaluation of Robetta models by PROCHECK, VERIFY 3D, ERRAT, and PROVE.
**TABLE S9.** Evaluation of AlphaFold models by PROCHECK, VERIFY 3D, ERRAT, and PROVE.
**TABLE S10.** Ramachandran distributions of Robetta 3D models of WxLIP proteins.
**TABLE S11.** Ramachandran distributions of 3D models of WxLIP proteins produced by AlphaFold.
**TABLE S12.** 3D‐ligand prediction of the binding site.
**TABLE S13.** Mass spectrometry analysis of pDUF916 tryptic digests.
**FIGURE S1.** Sequence alignment of 175 PGBD proteins from WxL clusters (Table S5). Sequences are identified using the GenBank code. Conserved residues (defined as residues invariant in at least 60 sequences) are indicated at the bottom of the alignment. The alignment was conducted using MUSCLE, and further organised using SeaView.
**FIGURE S2.** Sequence alignment of 175 HBD proteins from WxL clusters (Table S5). Sequences are identified using the GenBank code. Conserved residues (defined as residues invariant in at least 60 sequences) are indicated at the bottom of the alignment. The alignment was conducted using MUSCLE, and further organised using SeaView.
**FIGURE S3.** Structure prediction of WxLIP proteins by Phyre2. Structures (a–d) are respectively *Efm*WxLIP1, *Efm*WxLIP2, *Efm*WxLIP3, and *Efs*WxLIP. The predicted structures were obtained in April 2021.
**FIGURE S4.** Robetta prediction of WxLIP proteins. The proteins are the same as those shown in Figure S3. The predicted structures were obtained in April 2021.
**FIGURE S5.** Conserved residues in WxLIP. The comparisons are done between (a) proteins in the clusters of human symbionts from Figures 2 and 3, and (b) all PGBD/HBD proteins from bacterial genomes (Table S5). PGBD is in blue, the buttressing loop in maroon, HBD in red, and conserved residues in cyan. The most obvious difference is the lack of cyan residues at the top of (b).
**FIGURE S6.** Design of constructs for locus C domains. (a) Sequence alignment of WxLIP proteins. *Efm*WxLIP1, 2, and 3 represent Locus A, B, and C of *E. faecium* DO, and *Efs*WxLIP represents DUFE from *E. faecalis* V583. The sequence highlighted in yellow represents the signal peptide, dark blue is PGBD and cyan is HBD. Conserved residues are highlighted in red, sequences with one variation are highlighted in purple, and the highly conserved sequence NQIDK is boxed. β‐strand is shown as a blue arrow, the green spiral represents alpha helix, and the brown spiral represents the C‐terminal transmembrane helix. The sequences highlighted in red boxes indicate the starting and ending point of the three different constructs; SAS to NE is pDUF916 1, SAS to NN is pDUF916 2, and NE to NN is pDUF916 3. (b) Protein sequences. pDUF916 1, pDUF916 2, and pDUF916 3, with the 6 His‐Tag at the N‐terminus in sky blue followed by linker in bold. (c) Properties of the three constructs. The three constructs code for *Efm*WxLIP3 PGBD, *Efm*WxLIP3 PGBD and HBD, and *Efm*WxLIP3 HBD (i.e., DUF916, DUF916 + DUF3324, and DUF3324) respectively.
**FIGURE S7.** Schematic representation of the different *E. faecium* DO Locus C *Efm*WxLIP3 domain constructs in pOPINF vectors. The three vectors pDUF916 1_pOPINF, pDUF916 2_pOPINF, and pDUF916 3_pOPINF are shown; Green is respectively PGBD, PGBD + HBD, and HBD in the three constructs, with a His‐Tag on the Nterminal end in sky blue.
**FIGURE S8.** Analysis of supernatant from expression of *E. faecium* in BL21 (DE3) lemo. Lane 1: DUF916_2, 1 mM IPTG, 37°C, 4 h; Lane2: pDUF916_3, 1 mM IPTG, 37°C, 4 h; Lane 3: empty; Lane 4: unrelated protein; Lane 5: pDUF916_1, 0.5 mM IPTG, 25°C overnight; Lane 6: pDUF916_2, 0.5 mM IPTG, 25°C overnight; Lane 7: pDUF916_3, 0.5 mM IPTG, 25°C overnight; Lane 8: unrelated protein; Lane 9: pDUF916_1, 1 mM IPTG, 37°C, 4 h; Lane 10: pDUF916_2, 1 mM IPTG, 37°C, 4 h; Lane11: pDUF916_3, 1 mM IPTG, 37°C, 4 h; Lane 12: unrelated protein. The arrow indicates the expected position of DUF916_3 (18.2 kDa). DUF916_1 and DUF9196_2 are expected at 20.3 and 36.3 kDa respectively.

TABLE S5:

## Data Availability

All data are included in the manuscript and/or supporting information. Alphafold coordinates will be available on request to the corresponding author.
