# Supplementary material for: DUF916 and DUF3324 in the WxL protein cluster bind to WxL and link bacterial and host surfaces
Source: Protein Sci. 2023 Nov 1;32(11):e4806. doi: 10.1002/pro.4806 (PMC10599100; doi:10.1002/pro.4806)
Supplement: Supplementary file 1 — TABLE S1: Characteristics of WxLIP proteins from WxL clusters. TABLE S2. Position of different domains within different WxLIP proteins and detected by BlastP analysis and Motif Finder. TABLE S3. Position of different domains within different WxLIP proteins using InterPro, CDART, and SMART analysis. TABLE S4. Position of different domains within different WxLIP proteins by AlphaFold2 analysis. TABLE S6. Phyre2 analysis. TABLE S7. Robetta analysis of Enterococcus proteins having WxLIP proteins. TABLE S8. Evaluation of Robetta models by PROCHECK, VERIFY 3D, ERRAT, and PROVE. TABLE S9. Evaluation of AlphaFold models by PROCHECK, VERIFY 3D, ERRAT, and PROVE. TABLE S10. Ramachandran distributions of Robetta 3D models of WxLIP proteins. TABLE S11. Ramachandran distributions of 3D models of WxLIP proteins produced by AlphaFold. TABLE S12. 3D‐ligand prediction of the binding site. TABLE S13. Mass spectrometry analysis of pDUF916 tryptic digests. FIGURE S1. Sequence alignment of 175 PGBD proteins from WxL clusters (Table S5). Sequences are identified using the GenBank code. Conserved residues (defined as residues invariant in at least 60 sequences) are indicated at the bottom of the alignment. The alignment was conducted using MUSCLE, and further organised using SeaView. FIGURE S2. Sequence alignment of 175 HBD proteins from WxL clusters (Table S5). Sequences are identified using the GenBank code. Conserved residues (defined as residues invariant in at least 60 sequences) are indicated at the bottom of the alignment. The alignment was conducted using MUSCLE, and further organised using SeaView. FIGURE S3. Structure prediction of WxLIP proteins by Phyre2. Structures (a–d) are respectively EfmWxLIP1, EfmWxLIP2, EfmWxLIP3, and EfsWxLIP. The predicted structures were obtained in April 2021. FIGURE S4. Robetta prediction of WxLIP proteins. The proteins are the same as those shown in Figure S3. The predicted structures were obtained in April 2021. FIGURE S5. Conserved residues in W [file PRO-32-e4806-s001.pdf]

DUF916 and DUF3324 in the WxL protein cluster bind to WxL and link bacterial and host surfaces

Mahreen U Hassan, Roy R Chaudhuri and Mike P Williamson

Supplementary information

Table S1. Characteristics of WxLIP proteins from WxL clusters

WxLIP proteins															
	***Lp* WxLIP1**	***Lp* WxLIP2**	***Lp* WxLIP3**	***Lp* WxLIP4**	***Lp* WxLIP5**	***Lp* WxLIP6**	***Lp* WxLIP7**	***Lp* WxLIP8**	***Lp* WxLIP9**	***Lm* WxLIP1**	***Lm* WxLIP2**	***Efm* WxLIP1**	***Efm* WxLIP2**	***Efm* WxLIP3**	***Efs*WxLIP**
Number of amino acids	348	344	347	354	347	353	344	337	353	343	341	348	181	365	341
MWt (kDa)	38.5	37.6	38.0	38.8	38.5	39.3	38.5	37.4	39.6	38.8	38.6	39.2	20.2	41.6	38.6
Theoretical pI	10.03	10.57	9.97	10.26	10.08	10.10	10.09	9.89	10.45	8.55	9.07	5.18	6.84	5.21	5.24
Accession no	F9UNJ0	F9UQA7	F9USA9	F9USI4	F9USI9	F9USM8	F9UU92	F9UUC4	F9ULM1	Q8Y9H6	Q8Y9E4	A0A13 2Z4Q9	Q3XZ4 1	I3TYA2	Q830T9
Cluster	1	2	3	4	5	6	7	8	9	1	2	A	B	C	-
Previous name	DUFLB1	DUFLB2	DUFLB3	DUFLB4	DUFLB5	DUFLB6	DUFLB7	DUFLB8	DUFLB9	DUFLM1	DUFLM 2	DUFA	DUFB	DUFC	DUFE

Table S2. Position of different domains within different WxLIP proteins and detected by BlastP analysis and Motif Finder

**WxLIP proteins**														
**Domain position**	***Lp*** WxLIP1	***Lp*** WxLIP2	***Lp*** WxLIP3	***Lp*** WxLIP4	***Lp*** WxLIP5	***Lp*** WxLIP6	***Lp*** WxLIP7	***Lp*** WxLIP8	***Lp*** WxLIP9	***Lm*** WxLIP1	***Lm*** WxLIP2	***Efm*** WxLIP1	***Efm*** WxLIP2	***Efm*** WxLIP3	*Efs*WxLIP
**Signal peptide**	1-28	1-29	1-26	1-27	1-29	1-28	1-29	1-25	1-30	1-25	1-26	1-37	1-32	1-37	1-28
**PGBD**	35-180	38-185	37-180	46-189	34-175	34-174	39-182	30-171	43-186	31-173	33-179	38-180	37-180	38-180	29-174
**HBD**	190-307	195-305	191-304	199-313	185-299	184-298	192-308	181-296	196-310	183-299	190-300	190-304	-	190-323	184-301
**TMH**	-	-	311-344	314-352	300-344	305-347	311-343	307-335	-	300-341	303-340	305-344	-	-	303-340

Table S3. Position of different domains within different WxLIP proteins using InterPro, CDART, and SMART analysis

**WxLIP proteins**														
**Domain position**	***Lp*** WxLIP1	***Lp*** WxLIP2	***Lp*** WxLIP3	***Lp*** WxLIP4	***Lp*** WxLIP5	***Lp*** WxLIP6	***Lp*** WxLIP7	***Lp*** WxLIP8	***Lp*** WxLIP9	***Lm*** WxLIP1	***Lm*** WxLIP2	***Efm*** WxLIP1	***Efm*** WxLIP2	***Efm*** WxLIP3	*Efs*WxLIP
**Signal peptide**	1-26	1-23	1-20	1-25	1-24	1-28	1-29	1-25	1-30	1-25	1-26	1-31	1-32	1-35	1-19
**PGBD**	35-157	38-156	37-158	46-167	34-154	34-153	39-160	30-149	43-164	31-150	33-156	38-158	37-156	38-157	29-151
**HBD**	166-307	166-305	168-304	177-313	162-299	161-298	167-311	159-297	174-310	162-299	165-301	168-304	-	169-324	164-301
**TMH**	318-340	315-337	316-338	325-347	307-329	308-330	315-337	308-330	323-345	309-331	310-332	316-338	-	335-357	313-335

Table S4. Position of different domains within different WxLIP proteins by AlphaFold2 analysis

WxLIP proteins														
**Domain position**	***Lp*** WxLIP1	***Lp*** WxLIP2	***Lp*** WxLIP3	***Lp*** WxLIP4	***Lp*** WxLIP5	***Lp*** WxLIP6	***Lp*** WxLIP7	***Lp*** WxLIP8	***Lp*** WxLIP9	***Lm*** WxLIP1	***Lm*** WxLIP2	***Efm*** WxLIP 1	***Efm*** WxLIP 2	***Efm*** WxLIP 3	*Efs*WxLIP
**Signal peptide**	1-26	1-23	1-20	1-25	1-24	1-28	1-29	1-25	1-30	1-25	1-26	1-31	1-32	1-35	1-19
**PGBD**<br><br>**#amino acids**	35-182<br><br>147	38-183<br><br>145	37-186<br><br>149	46-193<br><br>147	34-182<br><br>148	34-182<br><br>148	39-186<br><br>147	30-183<br><br>153	41-192<br><br>151	31-181<br><br>150	31-181<br><br>150	38-186<br><br>148	37-181<br><br>144	38-186<br><br>148	29-188<br><br>159
**HBD**<br><br>**#amino acids**	191-308<br><br>117	191-305<br><br>114	191-306<br><br>115	201-313<br><br>112	191-301<br><br>110	191-301<br><br>110	191-312<br><br>121	187-302<br><br>115	201-313<br><br>112	188-299<br><br>111	191-301<br><br>110	191-305<br>114	-<br><br>-	191-324<br>133	191-301<br>110
**TMH**	318-346	314-342	314-347	322-354	307-346	308-352	315-343	306-336	318-352	307-342	310-340	316-346	-	333-365	311-335

Table S5 is a large file and is provided separately as an Excel sheet.

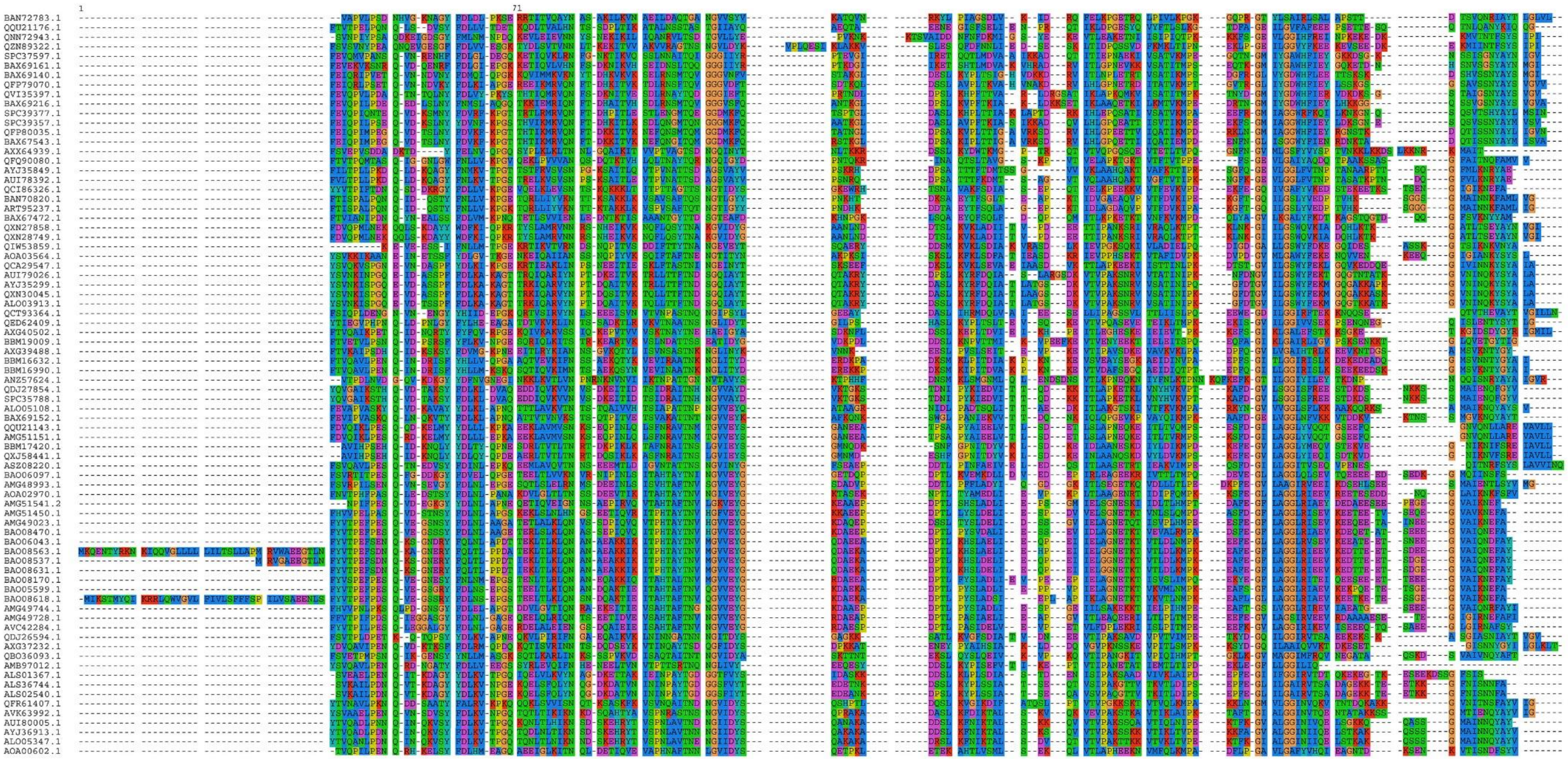

(Fig S1 cont)

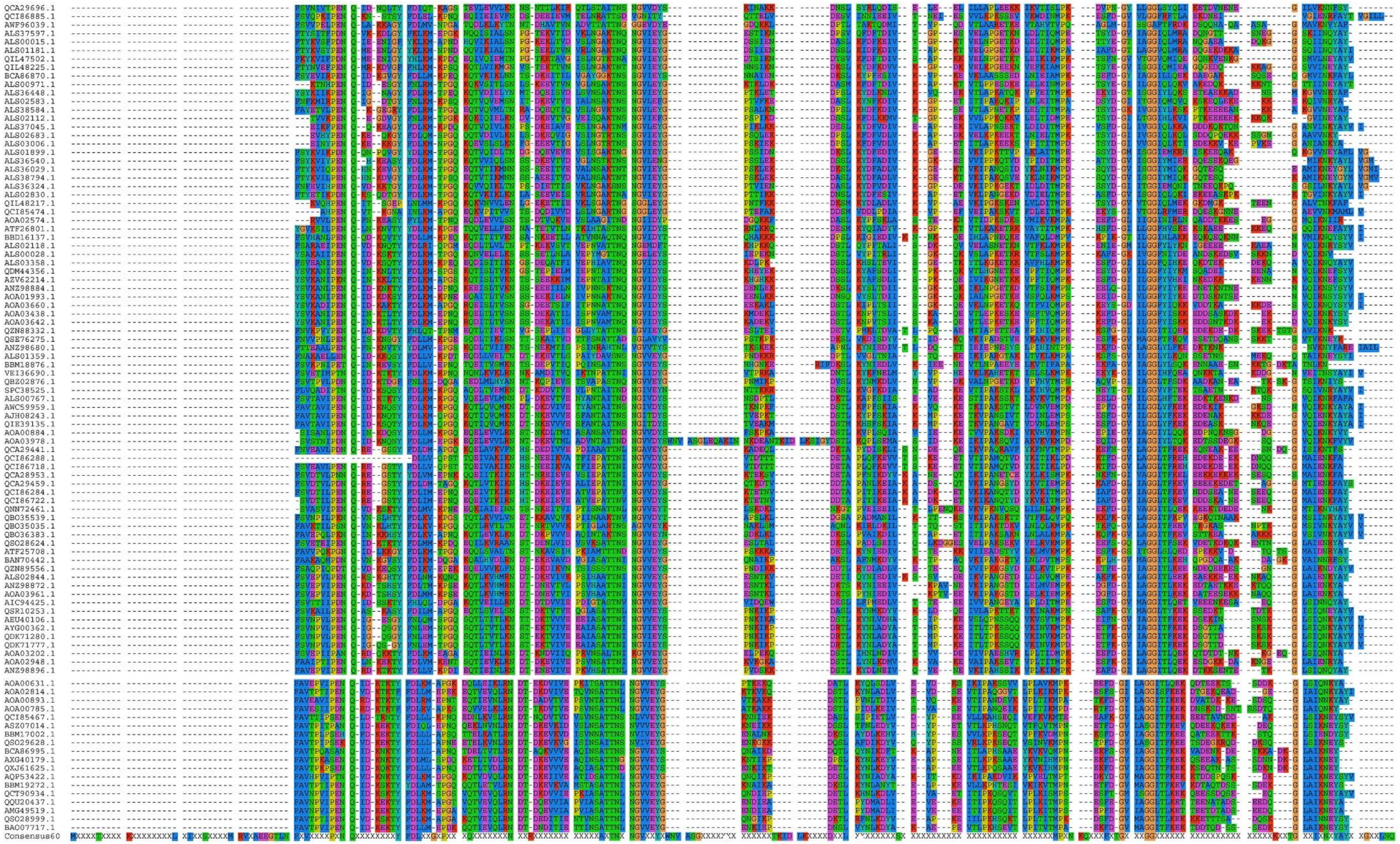

Fig S1. Sequence alignment of 175 PGBD proteins from WxL clusters (Table S5). Sequences are identified using the GenBank code. Conserved residues (defined as residues invariant in at least 60 sequences) are indicated at the bottom of the alignment. The alignment was conducted using MUSCLE, and further

organised using SeaView.

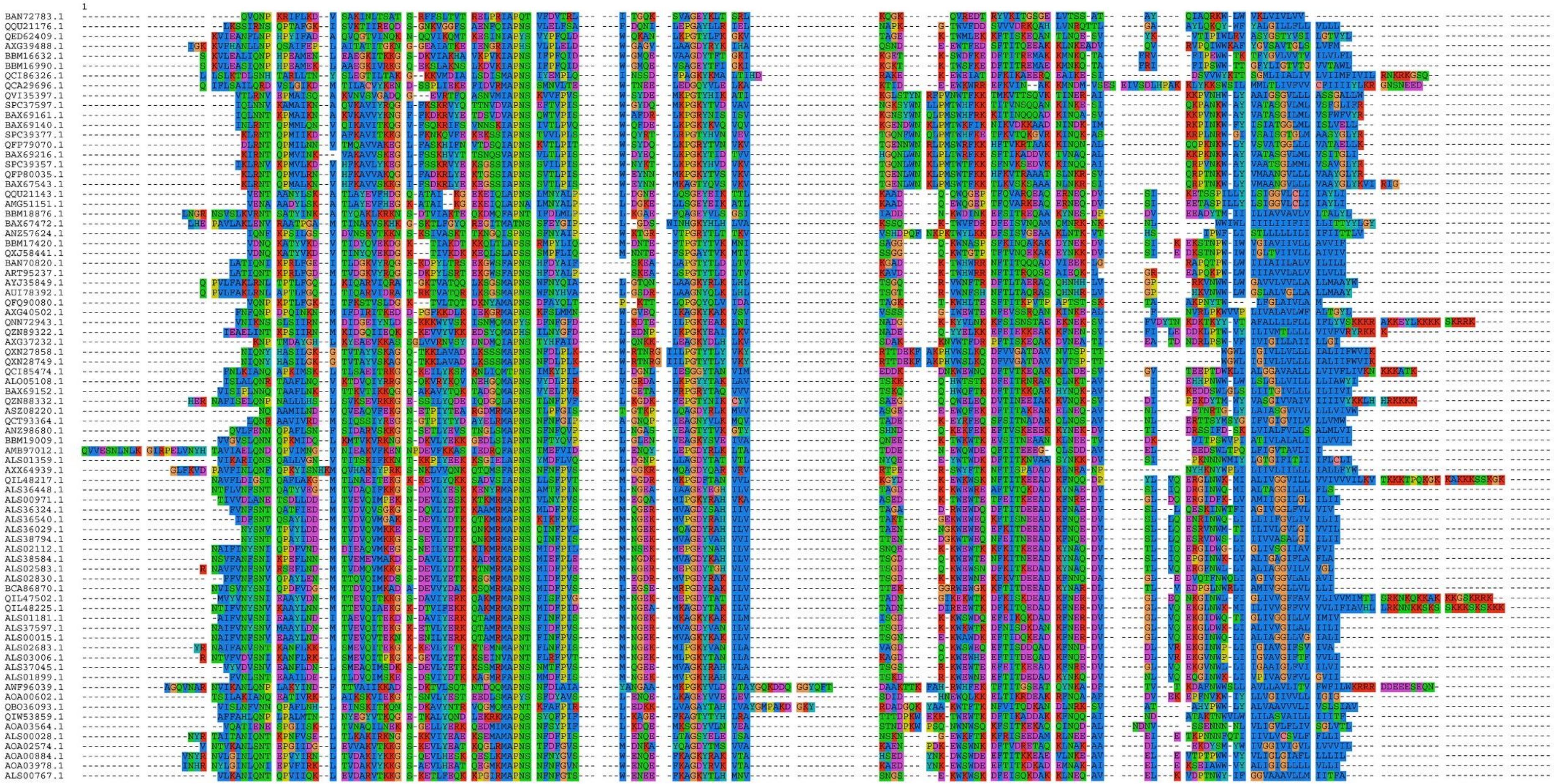

(Fig S2 cont)

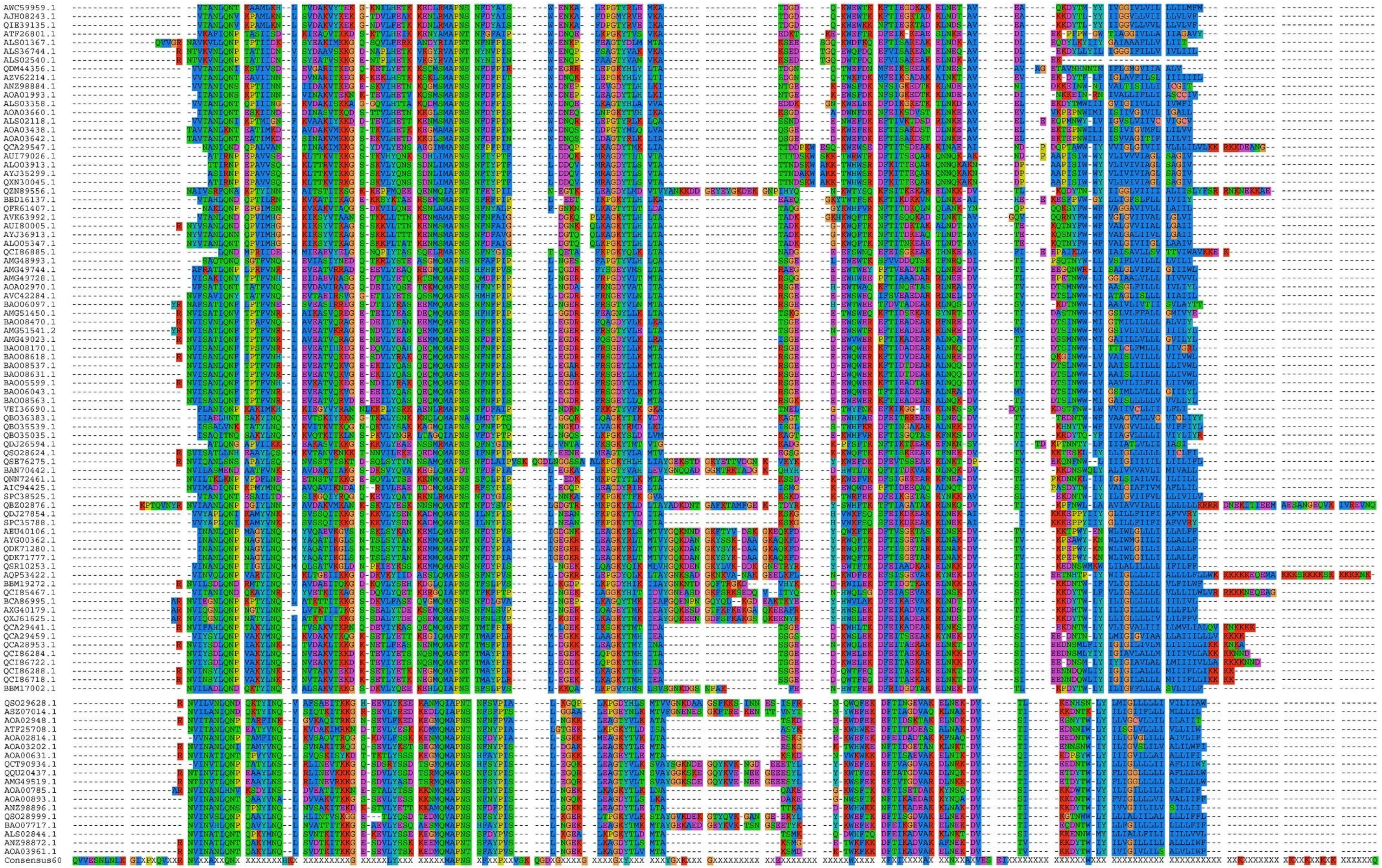

Fig S2. Sequence alignment of 175 HBD proteins from WxL clusters (Table S5). Sequences are identified using the GenBank code. Conserved residues (defined as residues invariant in at least 60 sequences) are indicated at the bottom of the alignment. The alignment was conducted using MUSCLE, and further organised using SeaView.

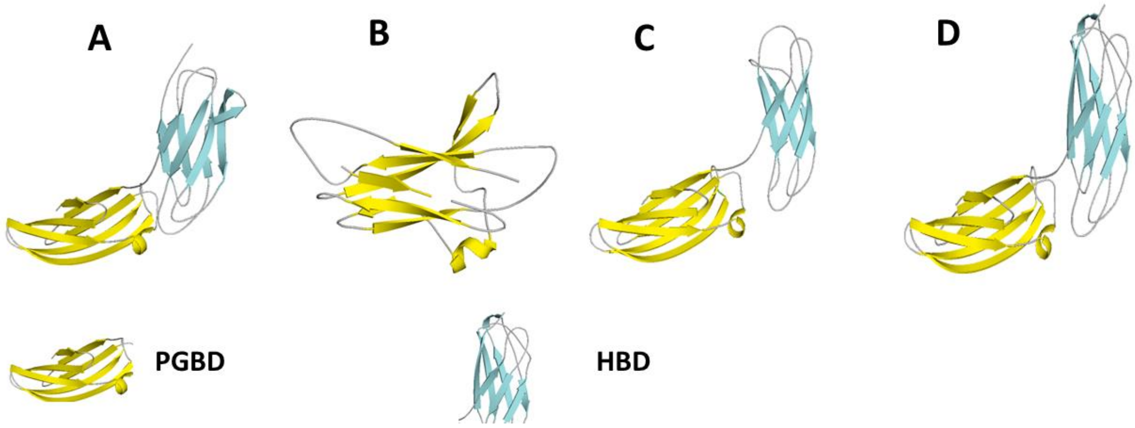

Figure S3. Structure prediction of WxLIP proteins by Phyre2. Structures A-D are respectively *Efm*WxLIP1, *Efm*WxLIP2, *Efm*WxLIP3 and *Efs*WxLIP. The predicted structures were obtained in April 2021.

Table S6. Phyre2 analysis

**Features**	***Efm*WxLIP1**	***Efm*WxLIP2**	***Efm*WxLIP3**	***Efs*WxLIP**
**Structure confidence**	98.8%	98.3%	98.8%	98.9%
**Protein Coverage**	59%	62%	52%	60%
**Disorder**	26%	29%	29%	27%
**Alpha Helix**	14%	17%	15%	16%
**Beta Sheets**	52%	48%	47%	50%
**TM Helix**	6%	9%	6%	6%
**Residue coverage**	56-296	56-180	56-285	47-293
**PGBD**	38-157	37-157	38-157	29-151
**Homology Model**	Protein-glutamine glutamyltransferase e3	Gram-negative pili assembly chaperone, N-terminal domain, Class 5 fimbriae chaperone cfaa	Protein-glutamine glutamyltransferase e3	Protein-glutamine glutamyltransferase e3

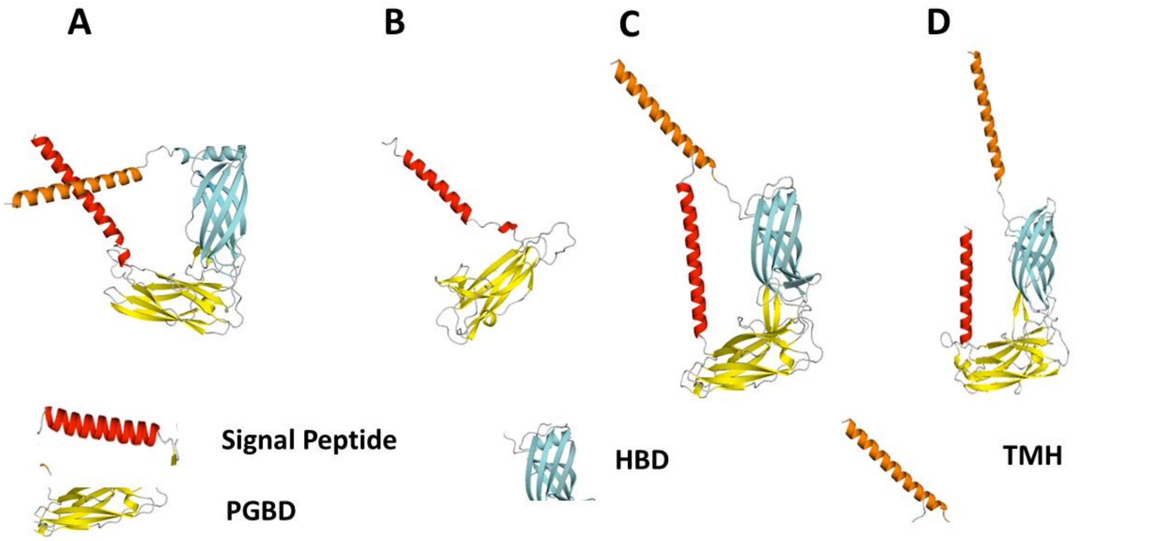

Figure S4. Robetta prediction of WxLIP proteins. The proteins are the same as those shown in Fig S3. The predicted structures were obtained in April 2021.

Table S7. Robetta analysis of *Enterococcus* proteins having WxLIP proteins

**Features**	***Efm*WxLIP1**	***Efm*WxLIP2**	***Efm*WxLIP3**	***Efs*WxLIP**
**Structure confidence**	69%	74%	72%	76%
**Protein Coverage**	100%	100%	100%	100%
**Residue coverage**	1-348	1-181	1-365	1-341
**Modelling Method**	TrRefine Rossetta			

Table S8. Evaluation of Robetta models by PROCHECK, VERIFY 3D, ERRAT and PROVE

**Protein Name**	**Procheck**				**Verify 3D**	**ERRAT**	**PROVE**
	**Core**	**Allowed**	**Generously**	**Disallowed**	**3D-ID score**	**Quality factor**	**Z-score**
***Efm*WxLIP1**	90.8%	7.3%	0.9 %	0.9 %	79.0%	90.3 %	0.26
***Efm*WxLIP2**	90.2%	9.2%	0.6 %	0.0 %	65.1%	97.4 %	0.25
***Efm*WxLIP3**	92.6%	6.5%	0.9%	0.0%	68.2%	88.6%	0.32
***Efs*WxLIP**	91.5%	7.6%	0.0%	0.30%	76.8%	87.5%	0.52

Table S9. Evaluation of AlphaFold models by PROCHECK, VERIFY 3D, ERRAT and PROVE

Protein Name	Procheck				Verify 3D	ERRAT	PROVE
	Core	Allowed	Generously	Disallowed	3D-ID score	Quality factor	Z-score
*Efm*WxLIP1	89.2%	10.4%	0.0%	0.3%	73.28%	91.5%	Fails
*Efm*WxLIP2	91.4%	8.6%	0.3%	0.3%	66.32%	95.5%	Fails
*Efm*WxLIP3	92.6%	6.5%	0.9%	0.0%	71.23%	94.0%	0.42
*Efs*WxLIP	90.5%	9.2%	0.0%	0.3%	75.3%	94.8%	0.52

Table S10. Ramachandran distributions of Robetta 3D models of WxLIP proteins

Protein	Most favored region	Additional allowed region	Generously allowed region	Disallowed region	Non-glycine and non-proline residues	End-residues (excl. Gly and Pro)	Glycine residues	Proline residues
*Efm*WxLIP1	287 (90.8%)	23 (7.3 %)	3 (0.9 %)	3 (0.9 %)	316 (100.0%)	02	14	16
*Efm*WxLIP2	147 (90.2%)	15 (9.2 %)	1 (0.6 %)	0 (0.0 %)	163 (100.0%)	02	9	7
*Efm*WxLIP3	312 (92.6%)	23 (6.8 %)	3 (0.9 %)	2 (0.6 %)	337 (100.0%)	02	14	12
*Efs*WxLIP	289 (91.5%)	24 (7.6 %)	2 (0.6 %)	1 (0.3 %)	316 (100.0%)	02	13	10

Table S11. Ramachandran distributions of 3D models of WxLIP proteins produced by AlphaFold

Protein	Most favoured region	Additional allowed region	Generously allowed region	Disallowed region	Non-glycine and non-proline residues	End-residues (excl. Gly and Pro)	Glycine residues	Proline residues
*Efm*WxLIP1	282 (89.2%)	33 (10.4 %)	1 (0.3 %)	0 (0.3 %)	220 (100.0%)	02	14	16
*Efm*WxLIP2	149 (91.4%)	14 (8.6 %)	0 (0.0 %)	0 (0.0 %)	163 (100.0%)	02	9	7
*Efm*WxLIP3	312 (91.4%)	22 (6.5 %)	3 (0.9 %)	0 (0.0 %)	337 (100.0%)	02	14	12
*Efs*WxLIP	286 (90.5%)	29 (9.2 %)	3 (0.0 %)	1 (0.3 %)	316 (100.0%)	02	13	10

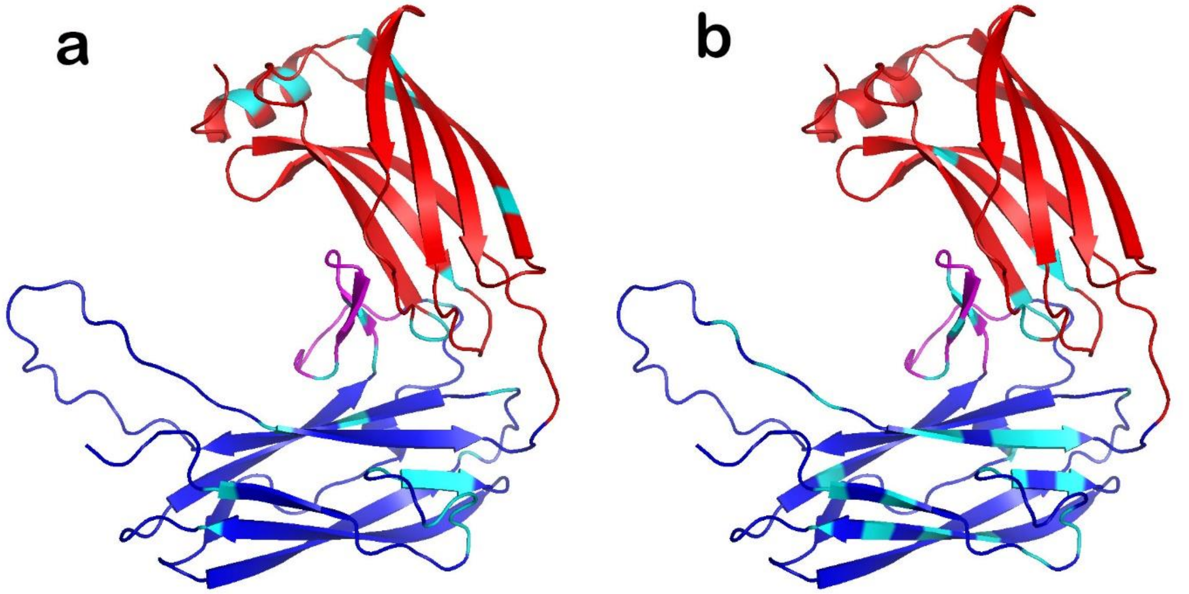

Figure S5. Conserved residues in WxLIP. The comparisons are done between (a) proteins in the clusters of human symbionts from Figures 2 and 3, and (b) all PGBD/HBD proteins from bacterial genomes (Table S5). PGBD is in blue, the buttressing loop in maroon, HBD in red, and conserved residues in cyan. The most obvious difference is the lack of cyan residues at the top of (b).

Table S12. 3D-ligand prediction of the binding site

Protein	Predicted binding site	Heterogen present in the predicted binding site
***Efm*WxLIP1**	THR^{134},ILE^{71},GLU^{69} and VAL^{43}	NAG4
***Efm*WxLIP2**	LYS^{114}	ZN
***Efm*WxLIP3**	ILE^{43},GLU^{67} ,LYS^{69} ,GLU^{71} and THR^{133}	NAG3
***Efs*WxLIP**	GLU^{34},GLU^{56},ILE^{58},LEU^{59},LYS^{60},VAL^{61},LYS^{112},ASP^{113},ALA^{127}, VAL^{128},GLU^{129},LEU^{130} and ARG^{131}	NAG40
***Lp*WxLIP1**	LYS^{39},ILE^{40},SER41,GLN66,ARG68,ALA118,ASN^{131},VAL^{133}	NAG5
***Lp*WxLIP2**	THR^{41}, LEU^{43}, PRO45, THR67, ARG69, SER71, ALA130	NAG7
***Lp*WxLIP3**	ARG^{70},TYR^{72},SER^{132}	NAG8
***Lp*WxLIP4**	ASN^{156},GLY^{157},VAL^{158},ILE^{198},VAL^{199},PRO^{200},GLN^{201},PRO^{202}, GLN^{203},THR^{228},ALA^{229},VAL^{230},HIS^{293},TRP^{295}	NAG1
***Lp*WxLIP5**	VAL^{39},ALA^{40},SER^{41},LEU^{64},ALA^{65},VAL^{66},LYS^{67},LYS^{129}, ILE^{130},VAL^{131},THR^{132}	NAG5
***Lp*WxLIP6**	THR^{190},ASN^{192}	NAG2
***Lp*WxLIP7**	LEU^{222},THR^{282},PHE^{287},TRP^{293}	CA20
***Lp*WxLIP8**	GLN^{33},LYS^{65},SER^{123},LYS^{125}	NAG6
***Lp*WxLIP9**	ASP^{112},GLN^{113},SER^{114},CYS^{202},ARG^{222},PRO^{224}	NAG2
***Lm*WxLIP1**	VAL^{186},LYS^{187},PRO^{188},GLU^{189},LYS^{214},ASP^{279},TRP^{281}	GAL4
***Lm*WxLIP2**	GLN^{188},ASN^{223},ASN^{279},GLY^{280},VAL^{281}	HEC20

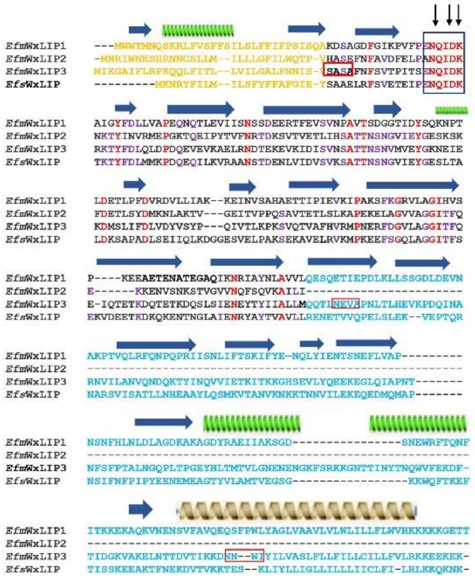

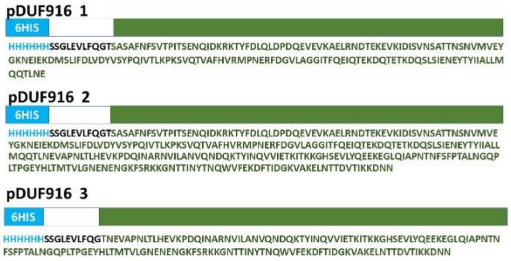

C

Domain	Molecular Weight (Da)	pI	Number of amino acids
pDUF916 1	20325.63	4.78	178
pDUF916 2	36324.57	5.13	320
pDUF916 3	18202.26	6.31	161

Figure S6. Design of constructs for locus C domains. (A) Sequence alignment of WxLIP proteins. *Efm*WxLIP1, 2 and 3 represent Locus A, B and C of *E. faecium* DO, and *Efs*WxLIP represents DUFE from *E. faecalis* V583. The sequence highlighted in yellow represents the signal peptide, dark blue is PGBD and cyan is HBD. Conserved residues are highlighted in red, sequences with one variation are highlighted in purple, and the highly conserved sequence NQIDK is boxed. β-strand is shown as a blue arrow, the green spiral represents alpha helix, and the brown spiral represents the C-terminal transmembrane helix. The sequences highlighted in red boxes indicate the starting and ending point of the three different constructs; SAS to NE is pDUF916 1, SAS to NN is pDUF916 2 and NE to NN is pDUF916 3. **(B) Protein sequences.** pDUF916 1, pDUF916 2 and pDUF916 3, with the 6 His-Tag at the N-terminus in sky blue followed by linker in bold. **(C) Properties of the three constructs.** The three constructs code for *Efm*WxLIP3 PGBD, *Efm*WxLIP3 PGBD and HBD, and *Efm*WxLIP3 HBD (ie DUF916, DUF916+DUF3324 and DUF3324) respectively.

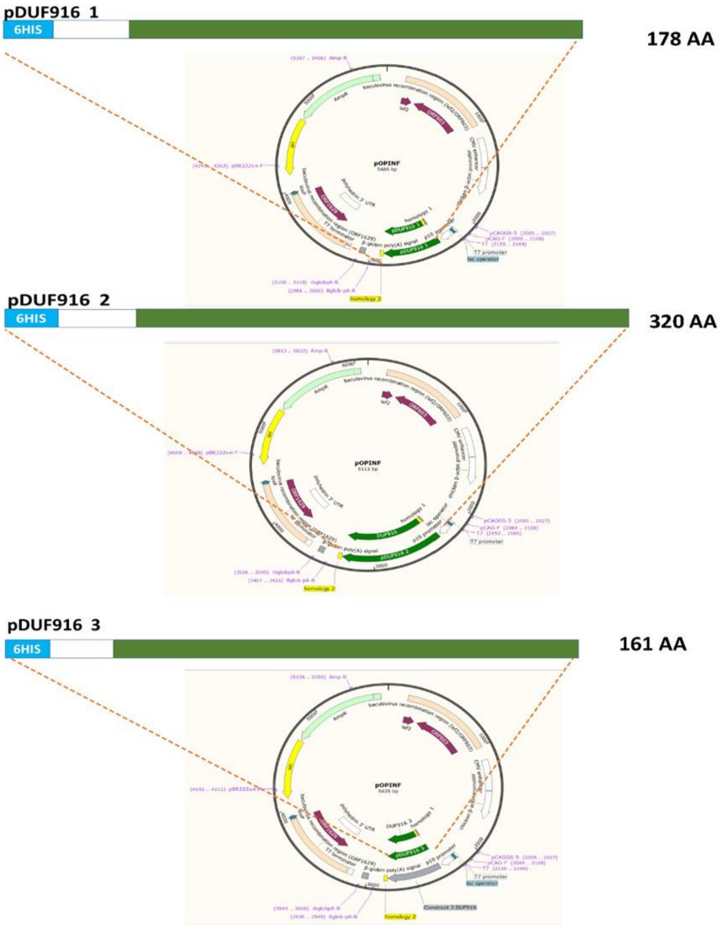

Figure S7. Schematic representation of the different *E. faecium* DO Locus C *Efm*WxLIP3 domain constructs in pOPINF vectors.

The three vectors pDUF916 1_pOPINF, pDUF916 2_ pOPINF and pDUF916 3_ pOPINF are shown; Green is respectively PGBD, PGBD+HBD, and HBD in the three constructs, with a His-Tag on the N-terminal end in sky blue.

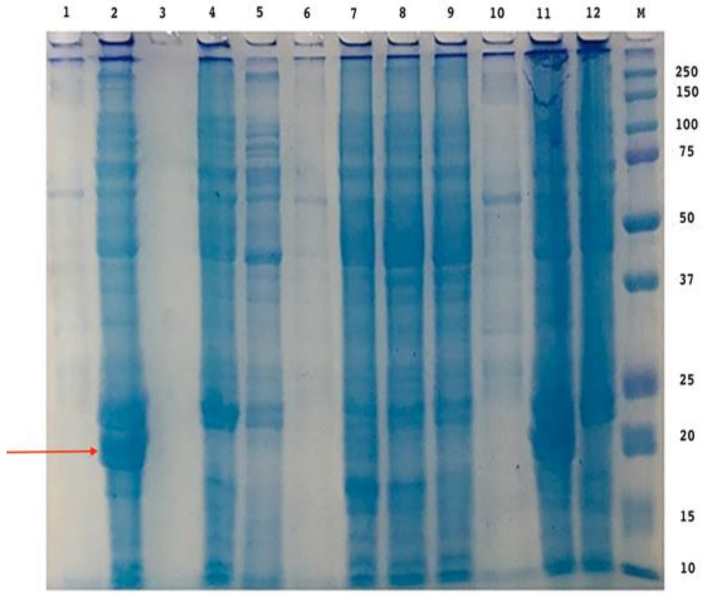

Figure S8. Analysis of supernatant from expression of *E. faecium* in BL21 (DE3) lemo. Lane 1: DUF916_2, 1mM IPTG, 37 °C, 4 hrs; Lane2: pDUF916_3, 1mM IPTG, 37 °C, 4hrs; Lane 3: Empty; Lane 4: unrelated protein; Lane 5: pDUF916_1, 0.5mM IPTG, 25°C overnight; Lane 6: pDUF916_2, 0.5 mM IPTG, 25°C overnight; Lane 7: pDUF916_3, 0.5 mM IPTG, 25°C overnight; Lane 8: unrelated protein; Lane 9: pDUF916_1, 1 mM IPTG, 37 °C, 4 hrs; Lane 10: pDUF916_2, 1 mM IPTG, 37°C, 4 hrs; Lane11: pDUF916_3, 1 mM IPTG, 37°C, 4 hrs; Lane 12: unrelated protein. The arrow indicates the expected position of DUF916_3 (18.2 kDa). DUF916_1 and DUF9196_2 are expected at 20.3 and 36.3 kDa respectively.

Table S13. Mass spectrometry analysis of pDUF916 tryptic digests.

construct	Unique peptide counts	Sequence coverage (%)	Protein score	iBAQ	Abundance (%)	sequence	Start-end	Peptide score
pDUF916_1	6	33.1	85	7.1 x 10^8	1.5	KTYFDLQLDPDQEVEVK	39-55	171
						TYFDLQLDPDQEVEVK	40-55	303
						AELRNDTEKEVK	56-67	67
						NDTEKEVK	60-67	73
						IDISVNSATTNSNVMVEYGK	68-87	206
						SVQTVAFHVR	116-125	98
pDUF916_2	3	9.4	24	1.5 x 10^7	0.03	NVILANVQNDQK	197-208	110
						TYINQVVIETK	209-219	113
						DFTIDGK	297-303	80
pDUF916_3	11	65	140	1.2 x 10^9	10.7	NVILANVQNDQK	38-49	208
						NVILANVQNDQKTYINQVVIETK	38-60	143
						TYINQVVIETK	50-60	215
						EGLQIAPNTNFSFPTALNGQPLTPGEYHLTMTVLGNENENGK	76-117	16
						KGNTTINYTNQWVFEK	122-137	57
						GNTTINYTNQWVFEK	123-137	101
						GNTTINYTNQWVFEKDFTIDGK	123-144	81
						VAKELNTTDVTIK	145-157	203
						ELNTTDVTIK	148-157	100
						ELNTTDVTIKK	148-158	28
						ELNTTDVTIKKDNN	148-161	69

iBAQ is a measure of the relative abundance of each protein. Abundance is the estimated proportion of total protein represented by the pDUF916 construct. The peptide score is a measure of uniqueness, with a larger score indicating higher confidence in the protein annotation.
